# Supplementary material for: Impact of Cassava Cultivars on Stylet Penetration Behavior and Settling of Bemisia tabaci Gennadius (Hemiptera: Aleyrodidae)
Source: Plants (Basel). 2024 Nov 15;13(22):3218. doi: 10.3390/plants13223218 (PMC11598763; doi:10.3390/plants13223218)
Supplement: Supplementary file 1 [file plants-13-03218-s001.zip › plants-3288933-supplementary.pdf]

**Supplements Materials**

**Impact of Cassava Cultivars on Stylet Penetration Behavior and Settling of *Bemisia tabaci* Gennadius (Hemiptera: Aleyrodidae)**

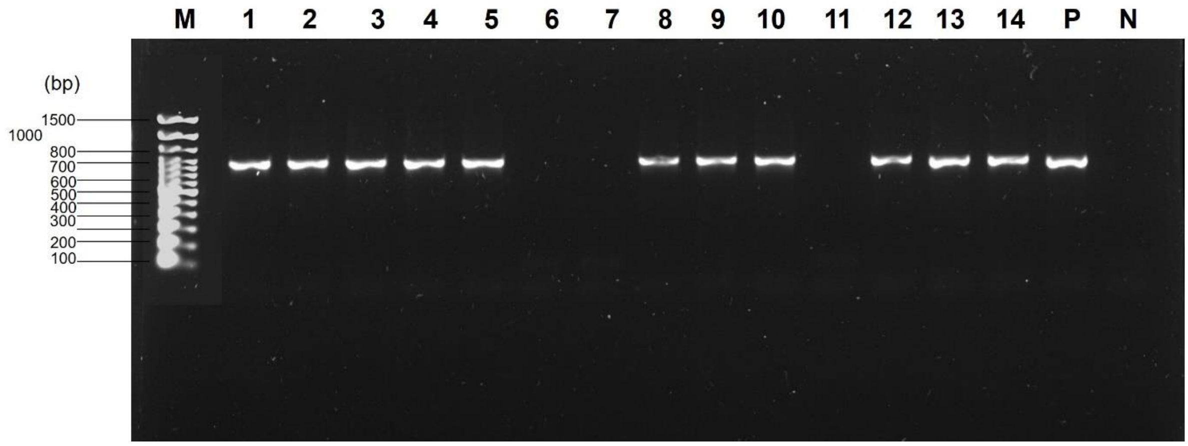


**Supplemental Figure S1.**

PCR products of the AV1 gene using SLCMV specific primers. DNA gel electrophoresis of PCR amplification from cassava samples

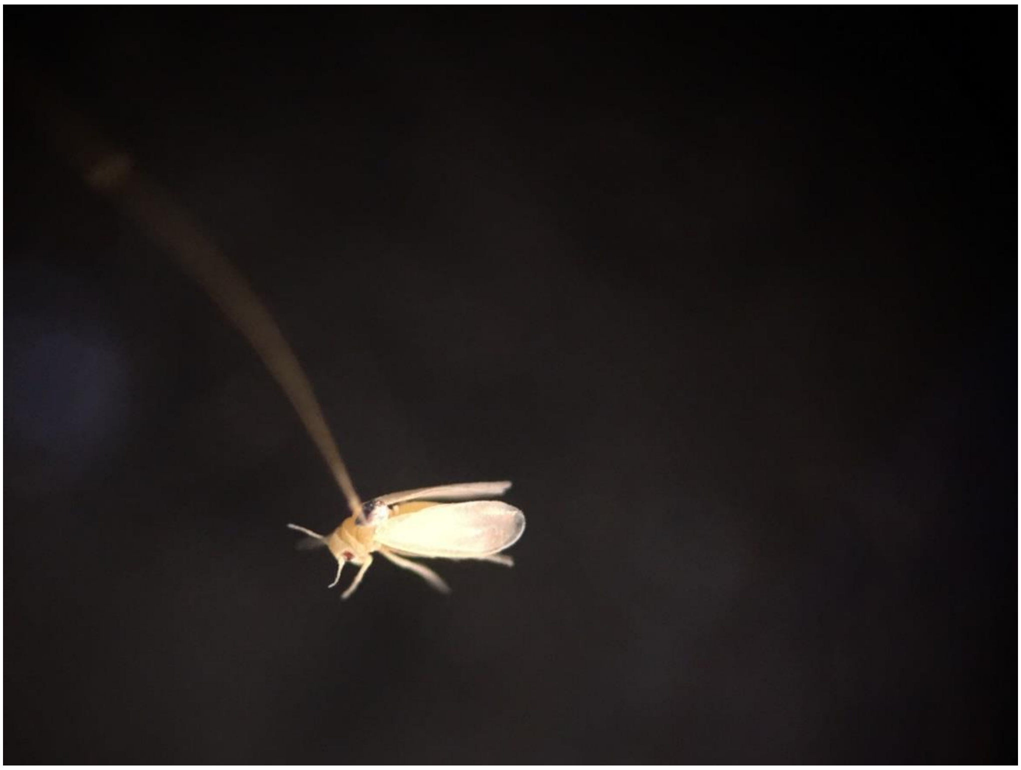

**Supplemental Figure S2.**
The adult whitefly was connected to a gold wire electrode (2.5 cm long, 12 μm diameter)**.**
